# Pyridinium 4-(trifluoro­meth­yl)benzene­sulfonate

**DOI:** 10.1107/S1600536809025835

**Published:** 2009-07-08

**Authors:** Gang Chen, Wei Xu, Zhen Yang, Zheng Fan

**Affiliations:** a College of Pharmaceutical Sciences, Zhejiang University of Technology, Hangzhou 310014, People’s Republic of China; b College of Biological & Environmental Engineering, Zhejiang University of Technology, Hangzhou 310014, People’s Republic of China

## Abstract

The title salt, C_5_H_6_N^+^·C_7_H_4_F_3_O_3_S^−^, is an ion pair in which the pyridium cation is linked to the 4-(trifluoro­meth­yl)benzene­sulfonate anion by an N—H⋯O hydrogen bond. The F atoms of the trifluoro­methyl group are disordered over two sites in a 0.584 (9):0.416 (9) ratio.

## Related literature

For the use of 4-(trifluoro­meth­yl)benzene­sulfonate anion as a rust inhibitor, see: Otomo (1993 ▶). For comparative bond dimensions for the anion, see: Bats *et al.* (1999 ▶); Bernhard *et al.* (1982 ▶); Kozioł & Podkowińska (1983 ▶), and for the cation, see: Djinović & Golič (1992 ▶) (1992); Ziemer & Rabis (2000 ▶).
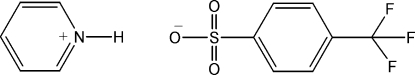


## Experimental

### 

#### Crystal data

C_5_H_6_N^+^·C_7_H_4_F_3_O_3_S^−^
*M* *_r_* = 305.27
Monoclinic, 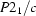

*a* = 5.7905 (5) Å
*b* = 9.0294 (7) Å
*c* = 24.988 (2) Å
β = 90.8220 (10)°
*V* = 1306.35 (18) Å^3^
*Z* = 4
Mo *K*α radiation
μ = 0.29 mm^−1^
*T* = 273 K
0.49 × 0.35 × 0.25 mm

#### Data collection

Bruker SMART APEX area-detector diffractometer
Absorption correction: multi-scan (*SADABS*; Bruker, 2000 ▶) *T*_min_ = 0.871, *T*_max_ = 0.931
6394 measured reflections
2291 independent reflections
1880 reflections with *I* > 2σ(*I*)
*R*_int_ = 0.031

#### Refinement

*R*[*F* ^2^ > 2σ(*F* ^2^)] = 0.088
*wR*(*F* ^2^) = 0.242
*S* = 1.07
2291 reflections
179 parameters
15 restraints
H-atom parameters constrained
Δρ_max_ = 0.70 e Å^−3^
Δρ_min_ = −0.53 e Å^−3^

### 

Data collection: *SMART* (Bruker, 2000 ▶); cell refinement: *SAINT* (Bruker, 2000 ▶); data reduction: *SAINT*; program(s) used to solve structure: *SHELXS97* (Sheldrick, 2008 ▶); program(s) used to refine structure: *SHELXL97* (Sheldrick, 2008 ▶); molecular graphics: *SHELXTL* (Sheldrick, 2008 ▶); software used to prepare material for publication: *SHELXL97*.

## Supplementary Material

Crystal structure: contains datablocks I, global. DOI: 10.1107/S1600536809025835/ng2593sup1.cif

Structure factors: contains datablocks I. DOI: 10.1107/S1600536809025835/ng2593Isup2.hkl

Additional supplementary materials:  crystallographic information; 3D view; checkCIF report

## Figures and Tables

**Table 1:** Hydrogen-bond geometry (Å, °)

*D*—H⋯*A*	*D*—H	H⋯*A*	*D*⋯*A*	*D*—H⋯*A*
N1—H1⋯O3	0.86	1.99	2.781 (6)	153

**Fig. 1.**
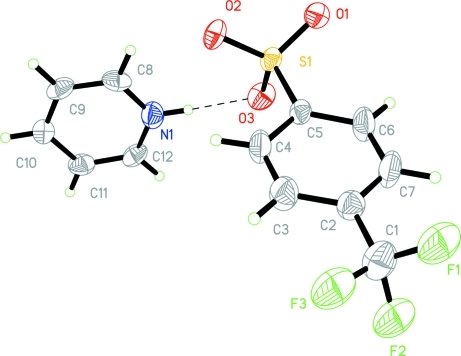
The cell unit of (I) with atom labels, showing 30% probability displacement ellipsoids. Parts of disorder F atoms was deleted for clarity.

**Fig. 2.**
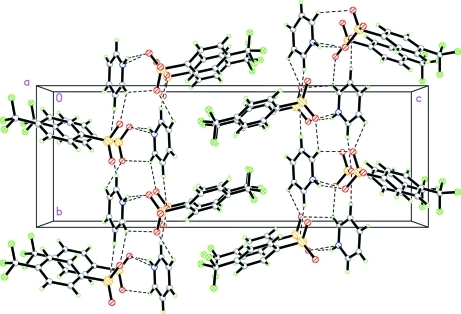
A packing diagram viewed down along the a axis. Hydrogen bonds are illustrated as thin lines. Parts of disorder F atoms was deleted for clarity.

## supplementary crystallographic information

### Comment

4-(Trifluoromethyl)benzenesulfonate was considered as excellent organic rust inhibitors which can effectively prevent corrosion of metals such as steel, copper, manganese and nickel (Otomo *et al.*, 1993). In our laboratory, a new compound containing 4-(trifluoromethyl)benzenesulfonate, (I), has been synthesized, its structure is studied hereafter.

Fig. 1 presents a view of the asymmetric unit: one pyridium cation and one 4-(trifluoromethyl)benzenesulfonate anion. In the anion the average C—C bond distance in the ring, 1.372 Å, is consistent with the value usually accepted. The internal angle (C4—C5—C6) is decreased to 118.0 (5)°, and endocyclic angle of C3—C2—C7 to 118.3 (5)°. The S—C distance of 1.795 (5)Å is close to the experimental values of 1.77 (Bats *et al.*, 1999), 1.766 (Bernhard *et al.*, 1982) and 1.782 (Kozioł & Podlowińska, 1983). The phenyl ring and the S atom are almost planar, which is displaced from the mean plane of the phenyl ring by 0.010 (6) Å. Three F atom in the trifluoromethyl group is disorder.

In the cation, distances in the pyridinium ring are in the range 1.319 (7)–1.372 (8)Å and angles 118.6 (5)–122.0 (5)°, which are similar to those found in research of Djinović & Golič (1992) and Ziemer *et al.* (2000).

The ion pairs are formed *via* a N1—H1···O3 hydrogen bond (Fig. 2). In addition, week C—H···O hydrogen bonds stabilize the crystal (Fig. 2 & Table 2).

### Experimental

4-(Trifluoromethyl)benzenesulfonic acid and pyridine in a molar ratio of 1:1 were mixed and dissolved in sufficient ethanol by heating to 365 K, when a clear solution resulted. Crystals of (I) were formed by gradual evaporation of excess ethanol over a period of one week at 293 K.

### Refinement

All of the H atoms were placed in calculated positions and allowed to ride on their parent atoms at distances of 0.86 (N—H), 0.93Å (aromatic), with *U*_iso_(H) = 1.2–1.5 *U*_eq_(C, N). Three disordered F atoms were split into approximately halves, with occupancies of 0.584 (9) and 0.416 (9). All the C—F distances are restrained to be identical with deviation of 0.01 Å.

### Crystal data

**Table:**

C_5_H_6_N^+^·C_7_H_4_F_3_O_3_S^−^	*F*(000) = 624
*M**_r_* = 305.27	*D*_x_ = 1.552 Mg m^−^^3^
Monoclinic, *P*2_1_/*c*	Mo *K*α radiation, λ = 0.71073 Å
Hall symbol: -P 2ybc	Cell parameters from 2733 reflections
*a* = 5.7905 (5) Å	θ = 2.4–25.0°
*b* = 9.0294 (7) Å	µ = 0.29 mm^−^^1^
*c* = 24.988 (2) Å	*T* = 273 K
β = 90.822 (1)°	Prism, colorless
*V* = 1306.35 (18) Å^3^	0.49 × 0.35 × 0.25 mm
*Z* = 4	

### Data collection

**Table:**

Bruker APEX area-detector diffractometer	2291 independent reflections
Radiation source: fine-focus sealed tube	1880 reflections with *I* > 2σ(*I*)
graphite	*R*_int_ = 0.031
φ and ω scans	θ_max_ = 25.0°, θ_min_ = 1.6°
Absorption correction: multi-scan (*SADABS*; Bruker, 2000)	*h* = −6→6
*T*_min_ = 0.871, *T*_max_ = 0.931	*k* = −10→10
6394 measured reflections	*l* = −22→29

### Refinement

**Table:**

Refinement on *F*^2^	Primary atom site location: structure-invariant direct methods
Least-squares matrix: full	Secondary atom site location: difference Fourier map
*R*[*F*^2^ > 2σ(*F*^2^)] = 0.088	Hydrogen site location: inferred from neighbouring sites
*wR*(*F*^2^) = 0.242	H-atom parameters constrained
*S* = 1.07	*w* = 1/[σ^2^(*F*_o_^2^) + (0.1117*P*)^2^ + 3.3401*P*] where *P* = (*F*_o_^2^ + 2*F*_c_^2^)/3
2291 reflections	(Δ/σ)_max_ = 0.001
179 parameters	Δρ_max_ = 0.70 e Å^−^^3^
15 restraints	Δρ_min_ = −0.53 e Å^−^^3^

### Special details

**Table:**

Geometry. All e.s.d.'s (except the e.s.d. in the dihedral angle between two l.s. planes) are estimated using the full covariance matrix. The cell e.s.d.'s are taken into account individually in the estimation of e.s.d.'s in distances, angles and torsion angles; correlations between e.s.d.'s in cell parameters are only used when they are defined by crystal symmetry. An approximate (isotropic) treatment of cell e.s.d.'s is used for estimating e.s.d.'s involving l.s. planes.
Refinement. Refinement of *F*^2^ against ALL reflections. The weighted *R*-factor *wR* and goodness of fit *S* are based on *F*^2^, conventional *R*-factors *R* are based on *F*, with *F* set to zero for negative *F*^2^. The threshold expression of *F*^2^ > σ(*F*^2^) is used only for calculating *R*-factors(gt) *etc*. and is not relevant to the choice of reflections for refinement. *R*-factors based on *F*^2^ are statistically about twice as large as those based on *F*, and *R*- factors based on ALL data will be even larger.

### Fractional atomic coordinates and isotropic or equivalent isotropic displacement parameters (Å^2^)

**Table:**

	*x*	*y*	*z*	*U*_iso_*/*U*_eq_	Occ. (<1)
S1	1.1078 (2)	0.88952 (12)	0.32068 (5)	0.0451 (4)	
O1	1.3546 (6)	0.8891 (4)	0.32268 (15)	0.0603 (10)	
O2	1.0094 (6)	1.0326 (4)	0.30758 (14)	0.0595 (10)	
O3	1.0162 (7)	0.7709 (4)	0.28780 (15)	0.0629 (10)	
C1	0.7591 (12)	0.7361 (7)	0.5413 (3)	0.086 (2)	
C2	0.8504 (10)	0.7742 (7)	0.4880 (2)	0.0598 (14)	
C3	0.7200 (11)	0.8601 (8)	0.4550 (3)	0.0756 (18)	
H3	0.5780	0.8954	0.4664	0.091*	
C4	0.7974 (10)	0.8958 (7)	0.4043 (2)	0.0679 (16)	
H4	0.7050	0.9535	0.3818	0.082*	
C5	1.0090 (8)	0.8472 (5)	0.38668 (19)	0.0444 (11)	
C6	1.1354 (10)	0.7576 (8)	0.4195 (2)	0.0730 (18)	
H6	1.2761	0.7203	0.4080	0.088*	
C7	1.0565 (12)	0.7217 (8)	0.4696 (3)	0.081 (2)	
H7	1.1455	0.6602	0.4915	0.098*	
F1'	0.9025 (16)	0.6547 (13)	0.5717 (3)	0.1146 (16)	0.584 (9)
F2'	0.5792 (17)	0.6419 (11)	0.5388 (4)	0.1146 (16)	0.584 (9)
F3'	0.700 (2)	0.8521 (9)	0.5715 (3)	0.1146 (16)	0.584 (9)
F1	0.921 (2)	0.7454 (19)	0.5793 (4)	0.1146 (16)	0.416 (9)
F2	0.672 (3)	0.5995 (12)	0.5458 (5)	0.1146 (16)	0.416 (9)
F3	0.577 (2)	0.8166 (15)	0.5579 (5)	0.1146 (16)	0.416 (9)
N1	0.6745 (8)	0.8321 (6)	0.21165 (18)	0.0650 (13)	
H1	0.7923	0.7923	0.2273	0.078*	
C8	0.6591 (10)	0.9796 (7)	0.2096 (2)	0.0610 (15)	
H8	0.7754	1.0376	0.2248	0.073*	
C9	0.4777 (10)	1.0437 (6)	0.1859 (2)	0.0613 (14)	
H9	0.4666	1.1464	0.1840	0.074*	
C10	0.3107 (10)	0.9570 (7)	0.1649 (2)	0.0628 (14)	
H10	0.1821	1.0001	0.1485	0.075*	
C11	0.3284 (10)	0.8056 (6)	0.1673 (2)	0.0619 (14)	
H11	0.2120	0.7459	0.1531	0.074*	
C12	0.5144 (10)	0.7456 (6)	0.1903 (2)	0.0581 (14)	
H12	0.5314	0.6432	0.1914	0.070*	

### Atomic displacement parameters (Å^2^)

**Table:**

	*U*^11^	*U*^22^	*U*^33^	*U*^12^	*U*^13^	*U*^23^
S1	0.0535 (8)	0.0315 (6)	0.0502 (7)	0.0055 (5)	−0.0052 (5)	0.0001 (5)
O1	0.057 (2)	0.053 (2)	0.071 (2)	0.0031 (17)	−0.0003 (17)	0.0060 (17)
O2	0.074 (3)	0.039 (2)	0.065 (2)	0.0168 (17)	−0.0059 (18)	0.0063 (16)
O3	0.086 (3)	0.044 (2)	0.058 (2)	−0.0024 (18)	−0.0059 (18)	−0.0113 (16)
C1	0.108 (6)	0.083 (5)	0.068 (4)	0.014 (4)	0.002 (4)	0.004 (4)
C2	0.064 (3)	0.061 (3)	0.055 (3)	0.000 (3)	−0.003 (2)	0.003 (3)
C3	0.067 (4)	0.086 (5)	0.074 (4)	0.025 (3)	0.014 (3)	0.013 (3)
C4	0.061 (3)	0.077 (4)	0.066 (3)	0.023 (3)	−0.006 (3)	0.015 (3)
C5	0.048 (3)	0.030 (2)	0.054 (3)	0.004 (2)	−0.009 (2)	−0.0026 (19)
C6	0.058 (3)	0.093 (5)	0.068 (4)	0.031 (3)	0.001 (3)	0.018 (3)
C7	0.079 (4)	0.096 (5)	0.068 (4)	0.031 (4)	−0.008 (3)	0.029 (4)
F1'	0.145 (4)	0.125 (4)	0.074 (2)	−0.006 (3)	0.011 (2)	0.022 (2)
F2'	0.145 (4)	0.125 (4)	0.074 (2)	−0.006 (3)	0.011 (2)	0.022 (2)
F3'	0.145 (4)	0.125 (4)	0.074 (2)	−0.006 (3)	0.011 (2)	0.022 (2)
F1	0.145 (4)	0.125 (4)	0.074 (2)	−0.006 (3)	0.011 (2)	0.022 (2)
F2	0.145 (4)	0.125 (4)	0.074 (2)	−0.006 (3)	0.011 (2)	0.022 (2)
F3	0.145 (4)	0.125 (4)	0.074 (2)	−0.006 (3)	0.011 (2)	0.022 (2)
N1	0.061 (3)	0.078 (3)	0.057 (3)	0.011 (3)	−0.002 (2)	0.013 (2)
C8	0.058 (3)	0.063 (4)	0.062 (3)	−0.025 (3)	−0.001 (2)	−0.008 (3)
C9	0.079 (4)	0.032 (3)	0.073 (4)	−0.006 (3)	0.012 (3)	0.000 (2)
C10	0.055 (3)	0.059 (3)	0.074 (4)	0.006 (3)	−0.002 (3)	0.010 (3)
C11	0.066 (4)	0.053 (3)	0.066 (3)	−0.018 (3)	−0.004 (3)	−0.003 (3)
C12	0.079 (4)	0.032 (3)	0.064 (3)	0.001 (2)	0.007 (3)	0.001 (2)

### Geometric parameters (Å, °)

**Table:**

S1—O1	1.429 (4)	C5—C6	1.358 (7)
S1—O3	1.446 (4)	C6—C7	1.378 (9)
S1—O2	1.447 (3)	C6—H6	0.9300
S1—C5	1.795 (5)	C7—H7	0.9300
C1—F1	1.330 (8)	N1—C12	1.319 (7)
C1—F2	1.337 (8)	N1—C8	1.336 (8)
C1—F1'	1.338 (7)	N1—H1	0.8600
C1—F3'	1.339 (7)	C8—C9	1.331 (8)
C1—F2'	1.345 (7)	C8—H8	0.9300
C1—F3	1.353 (8)	C9—C10	1.346 (8)
C1—C2	1.479 (9)	C9—H9	0.9300
C2—C3	1.354 (8)	C10—C11	1.372 (8)
C2—C7	1.370 (9)	C10—H10	0.9300
C3—C4	1.388 (8)	C11—C12	1.329 (8)
C3—H3	0.9300	C11—H11	0.9300
C4—C5	1.379 (7)	C12—H12	0.9300
C4—H4	0.9300		
			
O1—S1—O3	112.1 (2)	C6—C5—C4	118.0 (5)
O1—S1—O2	113.6 (2)	C6—C5—S1	120.3 (4)
O3—S1—O2	113.1 (2)	C4—C5—S1	121.5 (4)
O1—S1—C5	107.4 (2)	C5—C6—C7	120.4 (5)
O3—S1—C5	104.2 (2)	C5—C6—H6	119.8
O2—S1—C5	105.6 (2)	C7—C6—H6	119.8
F1—C1—F2	105.1 (9)	C2—C7—C6	121.7 (5)
F1'—C1—F3'	105.7 (7)	C2—C7—H7	119.1
F1—C1—F2'	127.7 (9)	C6—C7—H7	119.1
F1'—C1—F2'	98.8 (7)	C12—N1—C8	122.0 (5)
F3'—C1—F2'	108.6 (8)	C12—N1—H1	119.0
F1—C1—F3	107.1 (9)	C8—N1—H1	119.0
F2—C1—F3	100.0 (10)	C9—C8—N1	120.2 (5)
F1—C1—C2	111.7 (8)	C9—C8—H8	119.9
F2—C1—C2	115.5 (8)	N1—C8—H8	119.9
F1'—C1—C2	114.3 (6)	C8—C9—C10	118.6 (5)
F3'—C1—C2	115.0 (6)	C8—C9—H9	120.7
F2'—C1—C2	113.1 (6)	C10—C9—H9	120.7
F3—C1—C2	116.2 (7)	C9—C10—C11	120.6 (6)
C3—C2—C7	118.3 (5)	C9—C10—H10	119.7
C3—C2—C1	118.5 (5)	C11—C10—H10	119.7
C7—C2—C1	123.1 (5)	C12—C11—C10	119.0 (5)
C2—C3—C4	120.3 (5)	C12—C11—H11	120.5
C2—C3—H3	119.9	C10—C11—H11	120.5
C4—C3—H3	119.9	N1—C12—C11	119.6 (5)
C5—C4—C3	121.2 (5)	N1—C12—H12	120.2
C5—C4—H4	119.4	C11—C12—H12	120.2
C3—C4—H4	119.4		

### Hydrogen-bond geometry (Å, °)

**Table:**

*D*—H···*A*	*D*—H	H···*A*	*D*···*A*	*D*—H···*A*
N1—H1···O3	0.86	1.99	2.781 (6)	153
C4—H4···O1^i^	0.93	2.56	3.255 (7)	132
C8—H8···O2	0.93	2.47	3.192 (7)	136
C8—H8···O3^ii^	0.93	2.45	3.233 (8)	142
C9—H9···O1^ii^	0.93	2.43	3.274 (6)	151
C11—H11···O2^iii^	0.93	2.52	3.214 (8)	132
C12—H12···O1^iv^	0.93	2.42	3.324 (7)	166

Symmetry codes: (i) *x*−1, *y*, *z*; (ii) −*x*+2, *y*+1/2, −*z*+1/2; (iii) −*x*+1, *y*−1/2, −*z*+1/2; (iv) −*x*+2, *y*−1/2, −*z*+1/2.
